# Supplementary material for: The Mechanism of Acupuncture Regulating Autophagy: Progress and Prospect
Source: Biomolecules. 2025 Feb 11;15(2):263. doi: 10.3390/biom15020263 (PMC11852493; doi:10.3390/biom15020263)
Supplement: Supplementary file 1 [file biomolecules-15-00263-s001.zip › biomolecules-3443029-supplementary.pdf]

This study searched five databases, PubMed, Embase, Cochrane Library, Web of Science, and MEDLINE, from construction to March 2024. The flowchart of the search process is shown in Figure 1. Our online database search identified 310 potentially relevant papers, which were manually screened to identify relevant studies, resulting in the inclusion of a total of 109 papers.

- **Search strategy in each database**

| Database | Search strategy |
| --- | --- |
| PubMed | ((((((((((((((((((((((((((Acupuncture[MeSH Terms]) OR (Electroacupuncture[MeSH Terms])) OR (Acupuncture point[MeSH Terms])) OR (moxibustion[MeSH Terms])) OR (Acupuncture Therapy[MeSH Terms])) OR (Acupuncture, Ear[MeSH Terms])) OR (Artemisia[MeSH Terms])) OR (Acupuncture[Title/Abstract])) OR (Electroacupuncture[Title/Abstract])) OR (Manual acupuncture[Title/Abstract])) OR (Filiform needle[Title/Abstract])) OR (Acupuncture point[Title/Abstract])) OR (Acupoint[Title/Abstract])) OR (Auricular acupuncture[Title/Abstract])) OR (Warm acupuncture[Title/Abstract])) OR (Fire acupuncture[Title/Abstract])) OR (Pharmacoacupuncture[Title/Abstract])) OR (Acupotomy[Title/Abstract])) OR (scalp acupuncture[Title/Abstract])) OR (Moxibustion[Title/Abstract])) OR (Wormwood[Title/Abstract])) OR (Mugwort[Title/Abstract])) OR (Moxa[Title/Abstract])) OR (Acupuncture Therapy[Title/Abstract])) OR (Acupuncture, Ear[Title/Abstract])) OR (Artemisia[Title/Abstract])) AND (((((signal transduction[MeSH Terms]) OR (molecular[Title/Abstract])) OR (signal transduction[Title/Abstract])) OR (genetic[Title/Abstract])) OR (signaling pathway[Title/Abstract]))) AND ((((((((((((((((((((((Autophagy[MeSH Terms]) OR (Mitophagy[MeSH Terms])) OR (microautophagy[MeSH Terms])) OR (macroautophagy[MeSH Terms])) OR (chaperone-mediated autophagy[MeSH Terms])) OR (Autophagic Cell Death[MeSH Terms])) OR (autophagy (cellular[Title/Abstract]))) OR (Autophagy[Title/Abstract])) OR (Mitophagy[Title/Abstract])) OR (microautophagy[Title/Abstract])) OR (macroautophagy[Title/Abstract])) OR (chaperone-mediated autophagy[Title/Abstract])) OR (autophagy,cellular[Title/Abstract])) OR (cellular autophagy[Title/Abstract])) OR (autophagocytosis[Title/Abstract])) OR (reticulophagy[Title/Abstract])) OR (er phagy[Title/Abstract])) OR (nucleophagy[Title/Abstract])) OR (ribophagy[Title/Abstract])) OR (lipophagy[Title/Abstract])) OR (cell autophagy[Title/Abstract])) OR (Autophagic Cell Death[Title/Abstract])) |
| Embase | (((Acupuncture OR Electroacupuncture OR Acupuncture point OR moxibustion OR Acupuncture Therapy OR Acupuncture, Ear OR Artemisia OR Manual acupuncture OR Filiform needle OR Acupoint OR Auricular acupuncture OR Warm acupuncture OR Fire acupuncture OR Pharmacoacupuncture OR Acupotomy OR scalp acupuncture OR Wormwood OR Mugwort OR Moxa) AND (signal transduction OR signalling pathway OR genetic OR molecular) AND (Autophagic Cell Death OR Autophagy OR Mitophagy OR microautophagy OR macroautophagy OR chaperone-mediated autophagy OR autophagy, cellular OR cellular autophagy OR autophagocytosis OR reticulophagy OR er phagy OR nucleophagy OR ribophagy OR lipophagy OR cell autophagy OR autophagy cellular))) |
| Cochrane Library | (((Acupuncture OR Electroacupuncture OR Acupuncture point OR moxibustion OR Acupuncture Therapy OR Acupuncture, Ear OR Artemisia OR Manual acupuncture OR Filiform needle OR Acupoint OR Auricular acupuncture OR |

| | |
|---|---|
| | Warm acupuncture OR Fire acupuncture OR Pharmacoacupuncture OR Acupotomy OR scalp acupuncture OR Wormwood OR Mugwort OR Moxa) AND (signal transduction OR signalling pathway OR genetic OR molecular) AND (Autophagic Cell Death OR Autophagy OR Mitophagy OR microautophagy OR macroautophagy OR chaperone-mediated autophagy OR autophagy, cellular OR cellular autophagy OR autophagocytosis OR reticulophagy OR er phagy OR nucleophagy OR ribophagy OR lipophagy OR cell autophagy OR autophagy cellular))) |
| Web of Science | (TS=(((“Acupuncture” OR “Electroacupuncture” OR “Acupuncture point” OR “moxibustion” OR “Acupuncture Therapy” OR “Acupuncture, Ear” OR “Artemisia” OR “Manual acupuncture” OR “Filiform needle” OR “Acupoint” OR “Auricular acupuncture” OR “Warm acupuncture” OR “Fire acupuncture” OR “Pharmacoacupuncture” OR “Acupotomy” OR “scalp acupuncture” OR “Wormwood” OR “Mugwort” OR “Moxa”) AND (“signal transduction” OR “signalling pathway” OR “genetic” OR “molecular”) AND (“Autophagic Cell Death” OR “Autophagy” OR “Mitophagy” OR “microautophagy” OR “macroautophagy” OR “chaperone-mediated autophagy” OR “autophagy, cellular” OR “cellular autophagy” OR “autophagocytosis” OR “reticulophagy” OR “er phagy” OR “nucleophagy” OR “ribophagy” OR “lipophagy” OR “cell autophagy” OR “autophagy (cellular)”)))) AND LA=(“English”) |
| MEDLINE | (((Acupuncture OR Electroacupuncture OR Acupuncture point OR moxibustion OR Acupuncture Therapy OR Acupuncture, Ear OR Artemisia OR Manual acupuncture OR Filiform needle OR Acupoint OR Auricular acupuncture OR Warm acupuncture OR Fire acupuncture OR Pharmacoacupuncture OR Acupotomy OR scalp acupuncture OR Wormwood OR Mugwort OR Moxa) AND (signal transduction OR signalling pathway OR genetic OR molecular) AND (Autophagic Cell Death OR Autophagy OR Mitophagy OR microautophagy OR macroautophagy OR chaperone-mediated autophagy OR autophagy, cellular OR cellular autophagy OR autophagocytosis OR reticulophagy OR er phagy OR nucleophagy OR ribophagy OR lipophagy OR cell autophagy OR autophagy cellular))) |

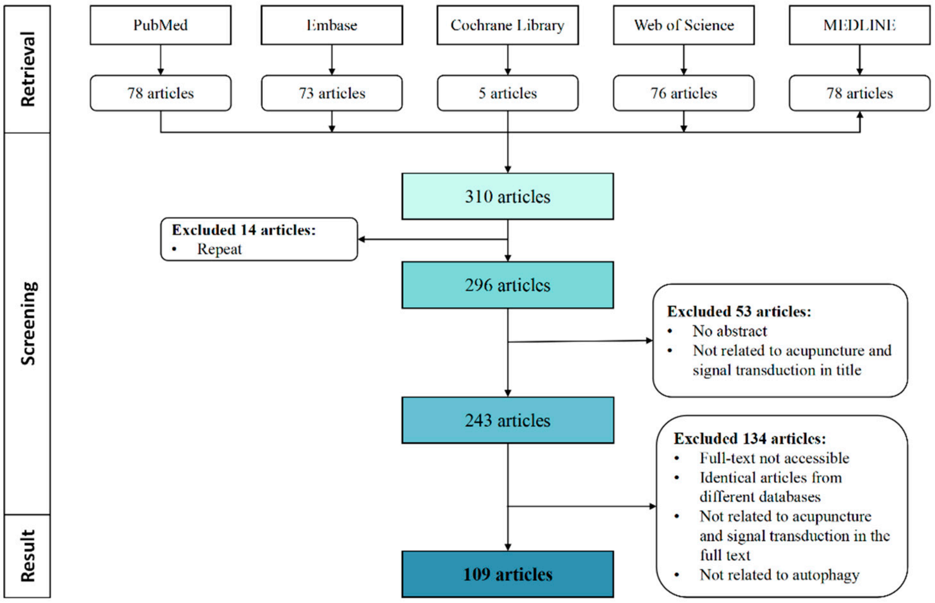


Figure S1. Flowchart of study selection
