# Supplementary material for: The Mechanism of Acupuncture Regulating Autophagy: Progress and Prospect
Source: Biomolecules. 2025 Feb 11;15(2):263. doi: 10.3390/biom15020263 (PMC11852493; doi:10.3390/biom15020263)
Supplement: Supplementary file 1 [file biomolecules-15-00263-s001.zip › biomolecules-3443029-Tables S1-S3.pdf]

Table S1. Acupuncture inhibits autophagy in the treatment of disease.

	Diseases	Location	Subjects	Acupoint	Intervention	Time of intervention	Molecular mechanism		Signal pathway/Regulator of autophagy	Reference
Nervous system	Ischemic Stroke	brain	SD rats	ST36,LI11	EA,1 mA,2 Hz	30min,QD for 3 days	↑:p62、LAMP-1、PI3K、P-mTOR/t-mTOR and AKT	↓:LC3I/II、CCAS3、LC3 and Atg7	PI3K/AKT/mTOR	[79]
		brain	SD rats	GV20,GV14,ST36	moxibustion	20min,QD for 3/5/7 days	↑:PI3K、p-PI3K、AKT and mTOR	↓:IL-1β、TNF-α、S-100β and NSE、	PI3K/AKT/mTOR	[83]
		brain	SD rats	LI11,ST36	EA,0.2 mA,1/20 Hz	30min,QD for 3 days	↑:mTORC1	↓:LC3BII/LC3BI、ULK1、Atg13 and Beclin1	mTORC1/ULK1/Beclin1	[80]
		brain	SD rats	GV20	EA,1 mA ,2/15 Hz	30min	↑:p62	↓:LC3II	Wnt/GSK3β	[158]
	Post-stroke depression	hippocampus	SD rats	GV14,GV26,GV20,GV24	acupuncture	40min,QD for 28 days	↑:PI3K、p-Akt and p-mTOR	↓:Beclin1、LC3B-Ⅱ and LC3B-Ⅱ/Ⅰ	PI3K/AKT/mTOR	[81]
	Central post-stroke pain	brain	SD rats	GV20,ST36	EA,1mA, 2 Hz/15 Hz	30min,QOD for 7 days	↑:p62	↓:LC3B-II/I、LAMP-1、COX-2 and β-catenin	COX-2/β-catenin	[82]
Respiratory system	Asthma	lung	C57BL/6 mice	GV14、BL13、ST36	acupuncture	20min,QOD for 14 days	↑:MAC、Th1 and Treg	↓:TGF-β、IL-4、IL-17、PERK、IRE-1、ATF6、Grp78 、LC3B、ATG5、EOS、NEU、LC3B、Th2 and Th17	ATG5	[85]
	Chronic obstructive pulmonary disease	lung	SD rats	ST36,BL13	EA,1-3 mA,4 Hz/20 Hz	30min,QOD for 14 days	↑:mTOR	↓:AMPK、ULK1、Beclin1、TNF-α and IL-6	AMPK/mTOR/ULK1	[87]
Circulatory system	Myocardial ischemia-reperfusion injury	heart	SD rats	PC6	EA,2 mA,2/100 Hz	20min,QD for 4 days	↑:SOD、mTOR/mTOR、GPX 4 and FTH 1	↓:ROS、MDA、ACSL4 and NCOA4	mTOR/ROS	[64]
	Chronic heart failure	heart	SD rats	BL13,BL15	moxibustion	20min,QD for 21 days	↑:p-mTOR/mTOR	↓:p53	mTOR	[91]

Digestive system	Functional dyspepsia	cells of Cajal	SD rats	ST36	EA,1mA,4 Hz	20min,QD for 7 days	↑:c-kit	↓:LC3Ⅱ/Ⅰ、Beclin 1、p-AMPK and p-ULK1	AMPK/ULK 1	[76]
	Crohn's disease	colon	SD rats	ST25,CV6	moxibustion	QD for 7 days	↑:SQSTM1/p62 and mTOR	↓:ATG16L1、NOD2、IRGM、LC3II、Beclin1、IL-1β、IL-17、TNF-β、PI3KC、LKB1 and AKT1	LKB1/mTOR /PI3KC	[93]
Other	Postherpetic neuralgia	bone marrow	SD rats	GB30、GB34	EA ,2 Hz	30min,QOD for 35 days	↑:p62、miR-133c、miR-486 and miR-223-3p	↓:LC3-II、ATG9、Rab1、miR-451-5p、miR-135a-5p and miR-7a-5p	ATG9	[96]
	Obesity	fat cell	Stat5fl/fl、Stat5NKO mice	ST36、ST44	EA	QD for 28 days	↑:miR 27 a	↓:ATG5 and ATG12	ATG 5/12	[97]
	Facial nerve injury	facial nerve	SD rats	ST4,ST6,TE17,LI4	EA,1mA,1-5 Hz	20min,QD for 14 days	↑:p62、GDNF、Rai、PI3K and mTOR	↓:Beclin1 and LC3	GDNF,PI3K/mTOR	[98]
	Vascular dementia	hippocampus	SD rats	GV20,GV14,BL23	EA,1 mA,10/50 Hz	30min,QD for 28 days		↓:NLRP3、Beclin1、LC3-Ⅱ/LC3-Ⅰ and ROS	ROS/NLRP3	[95]

Abbreviations: QD, daily; QOD, every other day.

Table S2. Activation of autophagy by acupuncture for the treatment of diseases.

	Diseases	Location	Subjects	Acupoint	Intervention	Time of intervention	Molecular mechanism		Signal pathway/Regulator of autophagy	Reference
Nervous system	Intracerebral hemorrhage	brain	SD rats	GV20,GB7	acupuncture	30min,TD for 7 days	↑:LC3、LC3II/I	↓:p62、p-mTOR and p-S6K1	mTOR	[102]
	Intracerebral hemorrhage	brain	SD rats	GV20,GB7	acupuncture	30min,QD for 15 days	↑: PINK1、Parkin、Beclin1 and NIX	↓:Caspase-9	PINK1/Parkin/NIX	[99]
	Intracerebral hemorrhage	brain	SD rats	GV20,GB7	EA ,1mA,4 Hz	15min,QD for 3、7 days	↑:Bcl-2	↓:p53、TOMM20、COX IV and caspase-3	p53 /Bcl-2	[100]

	Intracerebral hemorrhage	brain	SD rats	GV20,GB7	acupuncture	30min,QD for 1、3、7 days	↑:Beclin1、p-c-Jun and p-JNK	↓:LC3Ⅰ/Ⅱ	JNK	[101]
	Parkinson's disease	brain	C57BL6 mice	GB34,LR3	EA,1 mA,50 Hz	20min,QD for 5 days	↑:LC3、Beclin1、Atg7、Atg9A、Atg16L、Atg3、Atg7、LC3-II、DJ-1 and PINK1	↓:p62	PINK1	[103]
	Central Nervous System Diseases	cerebral cortex	SD rats	GV20,GV26	EA,3 mA,2/100 Hz	40 min		↓:TORC2 and Prr5l	TORC2/Prr5l	[105]
	Post-stroke cognitive impairment	hippocampus	SD rats	GV24,GV20	EA,0.5 mA,4/20HZ	30min,QD for 7 days	↑:PINK1、Parkin、LC3II/I and Beclin-1	↓:NLRP3、ROS、IL-1β and IL-18	PINK1/Parkin	[104]
	Alzheimer's disease	hippocampus	APP/PS1 mice	GV20、BL23	EA,1 mA,2 Hz	20min,QD for 15 days	↑:Beclin1、ATG5、ATG7、LC3II/I、AMPKα and AMPKβ1	↓:p62、mTOR and p70S6K	ATG5/7	[107]
	Alzheimer's disease	hippocampus	APP/PS1、C57BL/6J mice	GV20,GV16,GV14	moxibustion	20 min		↓:Aβ1-42、mTOR、p-mTOR、p70S6K and p-p70S6K	mTOR/p70S6K	[108]
Neurocognitive disorders	Alzheimer's disease	hippocampus	APP/PS1、C57BL/7J mice	GV20,GV14,GV16	moxibustion	20min,QD for 14 days	↑:LC3Ⅱ/Ⅰ	↓:Aβ1-42、p62 and p-P70S6K	mTOR/p70S6K	[159]
	Alzheimer's disease	hippocampus	SD rats	GV20,BL23	EA,1mA,50 Hz,	20min,QD for 48 days		↓:PHF-1、PI3K、AKT、p-AKT and mTOR	PI3K/AKT/mTOR	[109]
	Alzheimer's disease	hippocampus	APP/PS1 mice	GV20,GV14,GV16	moxibustion	15min,QD for 15 days	↑:LC3B-II/I and miR-511-3p	↓:PI3K、AKT3、mTOR、P62 and lncRNAs Six3os1	PI3K/AKT/mTOR	[110]
	Alzheimer's disease	hippocampus	C57BL/6J mice	GV20,GV14,GV16	moxibustion	20min,QD for 14 days		↓:PI3K、Akt、p-Akt、mTOR and p-mTOR	PI3K/AKT/mTOR	[111]
	Alzheimer's disease	hippocampus	APP/PS1 mice	GV20,BL23	EA,1 mA,2 Hz	20min,QD for 15 days	↑:Beclin1、ATG5、ATG7、LC3II/I、AMPKα and AMPKβ1	↓:p62、mTOR and p70S6K	AMPK/mTOR	[107]

	Alzheimer's disease	hippocampus	5xFAD mice	GV24,GB13	EA,0.3mA,2 Hz	15min,QD for 28 days	↑:CTSD、p-AMPKα（T172） and TFE3	↓:APP/CTFs、NLRP3、pro-/cleaved-CASP1、pro-/cleaved IL1B、ASC、NLRP3/ASC、SQSTM1 and ASC/CTSD	TFEB/TFE3	[112]
	Alzheimer's disease	hippocampus	APP/PS1 mice	GV20,KI1	EA,1 mA,1/50 Hz	15min,QD for 48 days	↑:LAMP1、TFEB and n-TFEB	↓:Aβ42、LC3II/LC3I、p62、pro-CTSD、pro-CTS and CTSD、	TFEB	[113]
	Alzheimer's disease	hippocampus	5XFAD mice	GV24,GB13	EA,0.3mA,2 Hz	15min,QD for 28 days	↑:TFEB、LAMP1、CTSD、LC3B-II and SQSTM1	↓:LC3B、SQSTM1、Fl-APP and CTF	TFEB	[114]
	Perioperative neurocognitive disorders	brain	SD rats	SP6,ST36	EA 1.5 mA,2/100 Hz,	30min,QD for 14 days	↑:PINK1、Parkin、LC3、Beclin1 and ROS	↓:IL-1β	PINK1/Parkin	[115]
Endocrine or metabolic diseases	Polycystic ovary syndrome	ovaries	SD rats	ST29,SP6	EA,0.8-1.3 mA ,2 Hz	15min/week 1,20min/week 2-3, 25min/week 4-5	↑:LC3、GLUT4、p-ERK、LC3II/I、Beclin1、PGC-1α and PPARγ	↓:p-mTOR、p-4E-BP1、GRP78、ATF4、CHOP、CYP17、CYP19 and p62	mTOR/4E-BP1	[116]
	Polycystic ovary syndrome	ovaries	SD rats	CV4,CV3,CV6, SP6,EX-CA1	acupuncture	30min,QD for 11 days	↑:E2	↓:LH、FSH、T、AMH、LncMEG3、PI3K、AKT、mTOR、p62 and LC3II/I	PI3K/AKT/mTOR	[117]
	Hypertriglyceridemia	liver	SD rats	ST36	moxibustion	QD for 10 weeks	↑:p-AMPK、p-ULK1、TFEB and LC3	↓:p-mTOR and p62	AMPK/mTOR	[5]
	Functional constipation	colon	Kunming mice	ST25,ST37	EA,1 mA,3/15 Hz	30min,QD for 14 days	↑:LC 3 and Beclin1	↓:PI 3 K、AKT and mTOR	PI3K/AKT/mTOR	[120]
Digestive system	Ulcerative colitis	colon	SD rats	ST25,ST37,RN12	EA,10 Hz/50 Hz	20min,QD for 5 days	↑:LC3B Ⅱ/LC3B Ⅰ、LC3B and p-AMPK/AMPK	↓:p62 and p-m TOR/mTOR	AMPK/mTOR	[121]
	Diabetic gastroparesis	cells of Cajal	SD rats	ST36,SP6,ST21	EA,2 mA,20/100 Hz	15min,QD for 21 days	↑:LC3 Ⅱ/ Ⅰ	↓:p62、PI3K、Akt、p-Akt and mTOR	PI3K/AKT/mTOR	[118]

Neoplasms	Colorectal cancer	colon	C57BL/6 mice	ST36,ST40	EA,2 Hz, 1 mA,	20 min, QD,three times a week for 11 weeks	↑:IL-10、SIRT1、Beclin1 and LC3	↓:IL-6、IL-17 and p62	SIRT1	[134]
	Colorectal cancer	colon	C57BL/6J mice	ST36,ST40	EA,2Hz,1 mA	20 min, QD,three times a week for 11 weeks	↑:IL-10、TNF-α、SIRT1、Beclin1、LC3 and Atg14	↓:IL-6、IL-17、p62 and miR-215	SIRT1/miR-215/Atg14	[133]
Diseases of the musculoskeletal system	Rheumatoid arthritis	synovium	SD rats	ST36	moxibustion	20min,QD for 15 days	↑:ULK1、Atg3、Atg5、Atg12、LC3II and Beclin1	↓: ESR、CRF、RF、IL-1β、IL-6、IL-23、IL-17、TNF-α、PI3K、Akt、mTOR、p-PI3K、p-Akt and p-mTOR	PI3K/AKT/mTOR	[7]
	Rheumatoid arthritis	synovium	Wistar rats	SP9,SP6	acupuncture	30min,QD for 21 days	↑:Beclin1 and LC3Ⅱ	↓:PI3K、AKT、p-AKT、mTOR and p-mTOR	PI3K/AKT/mTOR	[125]
	Rheumatoid arthritis	synovium	SD rats	ST36,CV4	moxibustion	20min,QD for 21 days	↑:AMPK、VPS34、ULK1、Atg13		AMPK/ULK1	[122]
	Rheumatoid arthritis	synovium	SD rats	ST36	moxibustion	20min,QD for 15 days	↑:ULK1、Atg3、Atg5、Atg12、LC3-Ⅱ and Beclin1		ULK1/ATG3/5/12	[123]
	Spleen deficiency	muscle	SD rats	ST36	EA,1～2 m A ,2 Hz/100 Hz	20min,QD for 10 days	↑:p-AMPK/AMPK、p-ULK1/ULK1 and LC3Ⅱ/Ⅰ		AMPK/ULK1	[75]
	Neurogenic cervical spondylosis	bone marrow	SD rats	GV14	moxibustion	QD for 7 days	↑:LC3、Act A、p-Smad2、p-Smad3、LC3II/I and Atg7		ATG7	[126]
	Neurogenic cervical spondylosis	bone marrow	SD rats	GV14	moxibustion	10min,QD for 7 days	↑:Beclin1 and Bcl-2		Bcl-2	[160]
	Neurogenic cervical spondylosis	bone marrow	SD rats	GV14	moxibustion	10min,QD for 7 days	↑:LC3	↓:Bax	Bax	[161]
	Neurogenic cervical spondylosis	bone marrow	SD rats	GV14	moxibustion	QD for 7 days	↑:LC3、Act A、p-Smad2、p-Smad3、LC3II/I and Atg7		Act A/Smads	[126]

	Neurogenic cervical spondylosis	bone marrow	SD rats	GV14	moxibustion	QD for 7 days	↑:Beclin1	↓:GRP78	Beclin1/GRP78	[127]
	Osteoarthritis of the knee	knee cartilage	SD rats	ST36,EX-LE4,ST35,GB34	EA,1mA,2Hz/100Hz	30min,QD for 28 days		↓:NLRP3、MMP-13 and TNF	NLRP3	[130]
	Osteoarthritis of the knee	knee cartilage	New Zealand rabbit	"Hedingci",Binneixia",Binwaixia",Chengfeijian",Chengfeijian",Weiyangci",Yinlingci"	needle knif	QD for 28 days	↑:Beclin1 and Bcl-2	↓:Caspase-3	Bcl-2、Caspase-3	[128]
	Osteoarthritis of the knee	knee cartilage	New Zealand rabbit		needle knif	QD for 21 days	↑:LC3II/I、PINK1 and Parkin	↓:p62	PINK1/Parkin	[129]
	Degenerative disc disease	bone marrow	SD rats		moxibustion	QD for 30 days	↑:LC3II/I、Beclin1、Bcl-2 and HIF-1α	↓:caspase-3、Bax and VEGF	HIF-1α/VEGF	[131]
	Aging	liver	SAMP8,SAMR1 mice	LR3,BL23	EA,1mA,2Hz	15min,QD for 14 days	↑:AMPK、p-AMPK、ULK1、p-ULK1、Atg5、Atg7、Atg13、Beclin1、ULK1 mRNA、HO-1 and SOD	↓:mTOR、p-mTOR and MDA	AMPK/mTOR/ULK1	[135]
Other	Aging	hippocampus	SD rats	GV20,ST36,LI4,LR3	acupoint catgut embedding	once a week	↑:RFP/GFP、LC3、PINK1 and PGC1-α	↓:p62	PINK1/Parkin	[136]

Abbreviations: TD, 2 times daily; QD, daily; QOD, every other day.

Table S3. Bidirectional regulation of autophagy by acupuncture for the treatment of diseases.

**Diseases**	**Location**	**Subjects**	**Acupoint**	**Intervention**	**Time of intervention**	**Molecular mechanism**	**Signal pathway/Regulator of autophagy**	**Results**	**Reference**

Cerebral ischemia/ reperfusion injury	brain	SD rats	GV20	EA,1-3 mA,4/20 Hz	30min	↑:P62、miR-275、WNT1 and β-catenin	↓:LC3、LC3II and miR-34	miR-34	Inhibiting autophagy	[144]
	brain	SD rats	CV12,CV4,ST36, ST40	EA,1 mA, 2/15 Hz	30min,QD for 5 days	↑:p62、SIRT1 and FOXO1	↓:LC3II/I、Ac-FOXO1 and Atg7	SIRT1/FOXO1	Inhibiting autophagy	[6]
	brain	SD rats	"Gan"(Liver)、"Shangjiao"(Upper-energizer)、"Xiajiao"(Lower-energizer)、"Shen"(Kidney)	acupuncture	30min,TD		↓:LC3B、Beclin1、ATF6 and XBP1	ATF6	Inhibiting autophagy	[162]
	brain	SD rats	GV20,GB7	EA, 2/20 Hz	30min,TD for 3 days	↑:p62 and Bcl-2	↓:LC3、LC3II/I、Beclin1、Bax、caspase-12、caspase-9、caspase-3、PARP、GRP78、ATF6、ATF4、CHOP、p-PERK/PERK、p-IRE1/IRE1 and p-eIF2α/eIF2α	PERK/IRE1/ATF6	Inhibiting autophagy	[163]
	brain	SD rats	GV20,LI4,LR3	EA, 1 mA ,2/20 Hz	30min,QD	↑:CX3CL1、ym1、fizz1 and arg1	↓:CX3CR1、NLRP3、INOS、TNFαand IL1β	CYLD/CX3CL1/NLRP3	Inhibiting autophagy	[124]
	hippocampus	SD rats	GV26,GV20	EA,1 mA,3 Hz/15 Hz	30min,QD for 5 days		↓:IL-6、IL-18、TNF-α、AMPK、Beclin1、VPS34、LC3B and LC3B-Ⅱ/Ⅰ	AMPK/Beclin-1/VPS34	Inhibiting autophagy	[143]
	brain	SD rats	GV20,GV24	EA,2 mA ,1/20 Hz	30min,QD for 8 days	↑:p-Akt、Beclin-1、PI3K and mTOR	↓:p53	PI3K/AKT	Activating autophagy	[140]
	brain	SD rats	GV14,GV20,GV26	acupuncture	30min,TD for 7 days	↑:PI3K、Beclin-1、 LC3B-Ⅱ/Ⅰ and Lamp2	↓:p62	PI3K/Beclin1	Activating autophagy	[141]
	brain	SD rats	LI11,ST36	EA,1mA,4/20 Hz	30min,QD for 3 days	↑:EA-6 h:LC3II/I、Beclin1;EA-12 h:LC3II/I、p-ERK1/2;EA-24 h:p62、LAMP1、p-ERK1/2、SIRT1、p-JNK	↓:EA-12 h:CCAS3;EA-24 h:LC3II/I、LC3、CCAS3	SIRT1/JNK/ERK	Bidirectional regulation	[142]
	hippocampus	SD rats	GV14,GV20,GV26	acupuncture	30min,TD for 7 days	↑:miR-34c-5p、Beclin-1、p62 and LC3B-Ⅱ/Ⅰ		miR-34c-5p	Bidirectional regulation	[145]

Myocardial ischemia	heart	SD rats	HT7,HT5	EA,1-2 mA,2 Hz	20min,QD for 7days	↑:p-Akt、p-Akt/Akt、p-mTOR and p-mTOR/mTOR		Akt/mTOR	Inhibiting autophagy	[146]
	heart	Wistar rats	PC6	EA,1 mA,2 Hz/15 Hz	30min,QD for 14 days		↓:AMPKa2	LKB1/AMPK/PFK2	Activating autophagy	[147]
Spinal cord injury	bone marrow	SD rats	GV14,GV4	EA,1 mA,2 Hz	20min,QD	↑:mTOR、p-PI3K/PI3K、AKT、p-mTOR/mTOR and p-p70S6/p70S6	↓:PTEN、Caspase-3 and p-PI3K	PI3K/AKT/mTOR	Inhibiting autophagy	[150]
	bone marrow	C57BL/6 mice	EX-B2	EA,1.5 Hz/7.5 Hz	QD for 7、14days	↑:LC3 II	↓:GRP78、Caspase12、p62 and CHOP	Caspase-12	Activating autophagy	[149]
	bone marrow	SD rats	EX-B2,ST36	EA,0.5 mA,2 Hz,	20min,QD for 7days	↑:Beclin1、LC3II/I	↓:miR-106b-5p、p62、Bax and Caspase-3	miR-106b-5p	Activating autophagy	[148]
Traumatic brain injury	brain	SD rats	GV20,GV26,ST36,PC6	EA,1 mA,2 Hz	15min,QD for 3、7、14days		↓:p-JNK and Beclin1	p-JNK/Beclin1	Inhibiting autophagy	[151]
	brain	SD rats	LI11,LI4,GV20,CV4,ST36,KI1	EA,1mA,1 Hz	15min,QD for 14days	↑:IL-10、p62 and mTOR	↓:LC3II、Beclin1、ATG5、ATG7、LAMP1、AMPK and TSC2	AMPK/mTOR	Inhibiting autophagy	[139]
	brain	SD rats	GV20,GV26,GV16,GV15,LI4	acupuncture	15min,QD for 3、7、14days	↑:three days later:LC3II/I、Beclin1;seven days later:p-mTOR/mTOR、p-ULK1ser757/ULK1;fourth days later:p62、p-mTOR/mTOR、p-ULK1ser757/ULK1	↓:three days later:p62、p-mTOR/mTOR、p-ULK1Ser757 /ULK1;seven days later:LC3、Beclin1;fourth days later:LC3、LC3II/I、Beclin1	mTOR/ULK1	Bidirectional regulation	[152]

Abbreviations: TD, 2 times daily; QD, daily.
